# Breast cancer survival in Nordic *BRCA2* mutation carriers—unconventional association with oestrogen receptor status

**DOI:** 10.1038/s41416-020-01056-4

**Published:** 2020-09-17

**Authors:** Elinborg J. Olafsdottir, Ake Borg, Maj-Britt Jensen, Anne-Marie Gerdes, Anna L. V. Johansson, Rosa B. Barkardottir, Oskar T. Johannsson, Bent Ejlertsen, Ida Marie Heeholm Sønderstrup, Eivind Hovig, Anne-Vibeke Lænkholm, Thomas van Overeem Hansen, Gudridur H. Olafsdottir, Maria Rossing, Jon G. Jonasson, Stefan Sigurdsson, Niklas Loman, Martin P. Nilsson, Steven A. Narod, Laufey Tryggvadottir

**Affiliations:** 1 Icelandic Cancer Registry, Icelandic Cancer Society, Reykjavik, Iceland; 2 Division of Oncology and Pathology, Department of Clinical Sciences, Lund University, Lund, Sweden; 3 Danish Breast Cancer Cooperative Group, Rigshospitalet, Copenhagen University Hospital, Copenhagen, Denmark; 4 Department of Clinical Genetics, Rigshospitalet, Copenhagen University Hospital, Copenhagen, Denmark; 5 Department of Medical Epidemiology and Biostatistics, Karolinska Institutet, Stockholm, Sweden; 6 Laboratory of Cell Biology, Department of Pathology, Landspitali University Hospital, Reykjavik, Iceland; 7 Faculty of Medicine, BMC, Laeknagardur, University of Iceland, Reykjavik, Iceland; 8 Department of Oncology, Landspitali University Hospital, Reykjavik, Iceland; 9 Department of Clinical Oncology, Rigshospitalet, Copenhagen University Hospital, Copenhagen, Denmark; 10 Department of Surgical Pathology, Zealand University Hospital, Roskilde, Denmark; 11 Department of Tumor Biology, Institute for Cancer Research, Radium Hospital, Oslo University Hospital, Oslo, Norway; 12 Department of Surgical Pathology, Zealand University Hospital, Slagelse, Denmark; 13 Center for Genomic Medicine, Rigshospitalet, Copenhagen University Hospital, Copenhagen, Denmark; 14 Faculty of Medicine, University of Iceland, Reykjavik, Iceland; 15 Department of Pathology, Landspitali University Hospital, Reykjavik, Iceland; 16 Cancer Research Laboratory, BMC, School of Health Sciences, University of Iceland, Reykjavik, Iceland; 17 Department of Hematology, Oncology and Radiation Physics, Skåne University Hospital, Lund, Sweden; 18 Womens’ College Research Institute, University of Toronto, Toronto, ON Canada

**Keywords:** Breast cancer, Prognostic markers

## Abstract

**Background:**

The natural history of breast cancer among *BRCA2* carriers has not been clearly established. In a previous study from Iceland, positive ER status was a negative prognostic factor. We sought to identify factors that predicted survival after invasive breast cancer in an expanded cohort of *BRCA2* carriers.

**Methods:**

We studied 608 women with invasive breast cancer and a pathogenic *BRCA2* mutation (variant) from four Nordic countries. Information on prognostic factors and treatment was retrieved from health records and by analysis of archived tissue specimens. Hazard ratios (HR) were estimated for breast cancer-specific survival using Cox regression.

**Results:**

About 77% of cancers were ER-positive, with the highest proportion (83%) in patients under 40 years. ER-positive breast cancers were more likely to be node-positive (59%) than ER-negative cancers (34%) (*P* < 0.001). The survival analysis included 584 patients. Positive ER status was protective in the first 5 years from diagnosis (multivariate HR = 0.49; 95% CI 0.26–0.93, *P* = 0.03); thereafter, the effect was adverse (HR = 1.91; 95% CI 1.07–3.39, *P* = 0.03). The adverse effect of positive ER status was limited to women who did not undergo endocrine treatment (HR = 2.36; 95% CI 1.26–4.44, *P* = 0.01) and patients with intact ovaries (HR = 1.99; 95% CI 1.11–3.59, *P* = 0.02).

**Conclusions:**

The adverse effect of a positive ER status in *BRCA2* carriers with breast cancer may be contingent on exposure to ovarian hormones.

## Background

*BRCA2* mutation carriers have a high lifetime risk of breast cancer. It is not well understood how the *BRCA2* mutation impacts on survival or the response to treatment. Breast cancers in *BRCA2* carriers are predominantly ER-positive, in contrast to *BRCA1* carriers, who mainly present with basal-like (ER-negative) breast cancers. In an Icelandic cohort study published in 2016, 285 *BRCA2* carriers diagnosed from 1935 to 2012 and 570 matched non-carriers were followed until 2014, and we showed that positive ER status was an adverse prognostic factor in *BRCA2* carriers.^1^ This was in contrast to the non-carriers, for whom ER-positive patients had superior survival. We sought to confirm these findings in a larger group of *BRCA2* carriers, with a range of pathogenic variants. We collected clinical information for *BRCA2* mutation carriers with invasive breast cancer from four Nordic countries (Denmark, Iceland, Norway and Sweden). Each centre contributed data on clinical presentation, treatment and outcome for breast cancer patients with a known pathogenic *BRCA2* variant from their institutions. The associations between prognostic factors, including ER status, tumour grade and various treatments, and death from breast cancer were investigated.

## Methods

Clinical information was retrieved by record linkage using the unique personal identification number assigned at birth to each citizen of the Nordic countries. The identification number is used in all contacts with the health system. Information was collected from health registries, including cancer registries, clinical registries, pathology laboratories and patient charts. When necessary, it was enriched by immunohistochemistry analyses of archived tissue specimens. We excluded women with invasive cancer at any site before breast cancer, except for non-melanoma skin cancer. We also excluded in situ breast cancer (DCIS) and patients with primary metastatic disease (Stage IV).

### Study populations

#### Denmark

*BRCA2* mutational screening was offered in Denmark as a diagnostic test from 1997 onwards. Clinical information about breast cancer diagnosis and treatment is available for Danish patients diagnosed from year 1977 and onwards from The Danish Breast Cancer Group (DBCG).^2^

Patients were included based on a positive family history of breast and/or ovarian cancer or early diagnosis of breast cancer or both breast and ovarian cancer in the patient. The national guidelines original stated that there should be at least 10% risk of detecting a pathogenic *BRCA*1/2 variant, but they were not always followed.

#### Iceland

The Icelandic carrier cases were identified from among 3577 women diagnosed with breast cancer in 1935–2012, tested for the *BRCA2* 999del5 founder mutation in the period 1995–2012 in the context of research projects. The majority was selected for *BRCA2* screening according to defined periods of diagnosis and year of birth, and only 0.3% were included on the basis of family history. For further description see ref. ^1^ For most of the patients in this historical population-based cohort, paraffin-embedded tumour specimens from pathology archives were used to establish mutation status. In the current study, we included all 187 carrier cases diagnosed in 1980 or later.

#### Norway

The Norwegian Radium Hospital invited unaffected women at high risk, for surveillance from 1988 onwards. Initially, this was based on family history, but later included women based on genetic test results. For the current study, incident breast cancer cases who were diagnosed in the follow-up period and carried a pathogenic *BRCA2* germline variant, and for whom the relevant variables were available, were included.^3^

#### Sweden

The majority of the Swedish participants (41 patients) came from a hereditary cancer clinic in Lund, where breast cancer patients with a positive family history for breast or ovarian cancer, young age at breast cancer diagnosis and/or contralateral breast cancer and/or ovarian cancer have been offered familial- and genetic counselling since year 1993. The remaining 21 carriers came from studies based on three population-based cohorts of breast cancer patients. All four cohorts have been described in previous publications.^4–7^

### Prognostic factors and treatment

For patients in all centres, information was requested on prognostic variables, method of breast cancer detection, date of diagnosis, date of *BRCA2* analysis and treatment. For 98% of the Danish group and 52% of the Icelandic group, ER status was indicated by percentage of cells staining positive by the immunohistochemical staining assay (IHC). The cut-off point used for positive was >1% for Denmark and >1% for Iceland. For the remaining women in those countries, only binary information (yes/no) was recorded. For Sweden and Norway, the cut-off point was 10%.

### Grouping of variants according to nucleotide position

Rebbeck et al. describe three groups of *BRCA2* mutations according to the association between nucleotide position and the ratio of breast vs ovarian cancer hazard ratios.^8^ Pathological variants were described using the Human Genome Variation Society nomenclature in which the nucleotide numbering is from the A of the ATG translation initiator codon. Multiple putative breast cancer cluster regions (BCCRs) were observed: BCCR1 spanning c.1 to c.596, BCCR1ʼ spanning c.772 to c.1806 and BCCR2 spanning c.7394 to c.8904. Two ovarian cancer cluster regions (OCCRs) were observed: OCCR1 spanning c.3249 to c.5681 and OCCR2 spanning c.6645 to c.7471. We grouped the Nordic mutations into three categories according to whether they were located in BCCRs, OCCRs or outside those cluster regions (“Other”).

### Statistical analysis

Chi-square test was used for comparing proportions of clinicopathological variables by ER status, and Chi-square trend test for proportions^9^ was used for assessing whether associations with age were statistically significant. All statistical tests were two-sided, and *P* values < 0.05 were considered to be statistically significant. In the survival analysis, patients were followed from the date of diagnosis of the first invasive breast cancer or date of sampling (*BRCA2* testing), whichever came last, until death or the last date of follow-up. Graphs were adjusted for competing risks of death by applying the user-written Stata command *stcompet*^10^ for non-parametric estimation of the breast cancer-specific cumulative incidence function, treating death due to other causes as a competing event.

Adjusted hazard ratios (HRs) were estimated by using Cox proportional hazard models. We used flexible parametric models, for checking and investigating violation of the proportional hazard assumption with baseline 5 df.^11^ The Norwegian carriers were not included in the survival analyses, as they lacked information on therapy other than surgery, and on cause of death. In the multivariable analysis, the hazard ratio was adjusted for size (T1, T2 and T3), lymph node metastases (yes/no), grade (2 and 3 vs 1), ER status (positive/negative), surgery (mastectomy/lumpectomy), oophorectomy (yes/no), prophylactic contralateral mastectomy (yes/no), chemotherapy (yes/no), radiation (yes/no) and hormone therapy (yes/no), country, mutation location and year of diagnosis. The variables neoadjuvant therapy and anti-HER2 therapy were not included in the survival analysis because only 16 women (3%) received each of those therapies.

Regarding missing values, we did sensitivity analyses; for each variable, we allocated a special code to unknown values and compared the survival curve for this specific category with curves for other categories. We then allocated the missing variable to the category for which the curve was most similar, e.g., for lymph node status, the unknowns were converted to a positive status. Neither leaving out unknowns, nor including them this way, changed the results in sensitivity analyses as compared to allocating a separate code for unknowns, except for grade, for which unknown was kept as a separate category.

To avoid survivorship bias, we used left-truncated survival analysis, that is, the follow-up time began at the date of sampling (*BRCA2* validation) for all women who were genetically tested using a blood sample drawn after diagnosis. To avoid immortal person-time bias,^12^ bilateral oophorectomy and contralateral prophylactic mastectomy were included in the multivariate analysis as time-dependent covariates, e.g., the time at risk considered them as a time-dependent variable with value 0 before oophorectomy and 1 after. To avoid potential selection bias resulting from the fact that women with a more favourable disease development might be prioritised for those operations, we considered only oophorectomies and prophylactic mastectomies occurring within 2 years from diagnosis (early oophorectomies). Operations occurring thereafter were treated as if they had not occurred. We compared women who had an early oophorectomy with those who did not, to check for selection bias that might affect the survival analyses.

As the proportional hazard assumption was not fulfilled for ER status (Grambsch–Therneau test, i.e., test of nonzero slope of Schoenfeld residuals vs time), we divided the follow-up interval into 0–5 years and 5+ years. By including an interaction term between ER status and the respective variables in the Cox model and using the Wald test, we tested in separate models for interactions with menopausal status (age ≤ 50 years vs 51+ years), bilateral oophorectomy, endocrine therapy, chemotherapy and mutation location. All analyses were performed using STATA Statistical Software Stata/IC 14.1 for Windows.

## Results

This study population consisted of 608 breast cancer patients with a pathogenic *BRCA2* variant, diagnosed between 1975 and 2018 (Table 1). The median follow-up time was 9.8 years. There were 379 premenopausal cases (i.e., ≤ 50 years at diagnosis) and 229 postmenopausal cases.

**Table 1:** Baseline characteristics and treatment among Nordic *BRCA2* carriers according to country.

	Denmark	Iceland	Norway	Sweden	Total
Number of invasive cases	335	187	24	62	608
Year at dx, median [range]	2005 [1977–2018]	1994 [1980–2012]	2006 [1994–2012]	2002 [1975–2016]	2001 [1975–2018]
Exit year, final check of life status	2019	2017	2014	1991–2019	1991–2019
Years of follow-up, median [range]	10.1 [1–37]	11.2 [1–38]	6.3 [2–20]	8.3 [1–37]	9.8 [1–38]
Age at dx, median [range]	45 [24–91]	48 [29–82]	52 [30–74]	46 [27–86]	46 [24–91]
Number of deaths	72 (21%)	116 (62%)	3 (13%)	25 (40%)	216 (36%)
BC deaths	45	78	–	19	142
Unknown cause	1	0	3	0	4
Timing of genetic testing	335	187	24	60	606
Before or at dx	62 (18%)	136 (73%)	24 (100%)	9 (15%)	231 (38%)
Within 2 y after dx	127 (38%)	34 (18%)	0 (0%)	26 (43%)	187 (31%)
>2 y after dx	146 (44%)	17 (9%)	0 (0%)	25 (42%)	188 (31%)
Unknown	0	0	0	2	2
Morphology					
Ductal	287 (87%)	160 (86%)	–	48 (92%)	495 (87%)
Lobular	24 (7%)	19 (10%)	–	4 (8%)	47 (8%)
Other	18 (5%)	8 (4%)	–	0	26 (5%)
Unknown	6	0	62	10	78
Size in mm, median [range]	19 [1–120]	20 [1–110]	10 [0–51]	20 [4–51]	20 [0–120]
Unknown	5	14	1	1	21
Lymph node positive	173 (53%)	94 (52%)	5 (25%)	32 (52%)	304 (52%)
Unknown	8	6	4	1	19
Grade 1	39 (13%)	20 (11%)	3 (14%)	2 (5%)	64 (11%)
2	138 (44%)	85 (47%)	12 (57%)	13 (33%)	248 (45%)
3	135 (43%)	76 (42%)	6 (29%)	25 (62%)	242 (44%)
Unknown	23	6	3	22	54
Oestrogen-receptor positive	253 (79%)	136 (74%)	15 (75%)	43 (78%)	447 (77%)
Unknown	15	4	4	7	30
Her2 positive	20 (7%)	4 (10%)	–	3 (10%)	27 (8%)
Unknown	48	146	24	32	250
Bilateral oophorectomy	254 (76%)	48 (26%)	20 (83%)	36 (58%)	358 (59%)
Before BC dx	16 (6%)	8 (17%)	14 (70%)	1 (3%)	39 (11%)
≤2 years after dx	122 (48%)	13 (27%)	5 (25%)	16 (44%)	156 (44%)
>2 years after dx	116 (46%)	27 (56%)	1 (5%)	19 (53%)	163 (45%)
Surgery at diagnosis					
Lumpectomy	143 (44%)	48 (26%)	6 (29%)	16 (26%)	213 (36%)
Mastectomy	185 (56%)	138 (74%)	15 (71%)	46 (74%)	384 (64%)
Unknown	7	1	3	0	11
Adjuvant chemotherapy					
None	88 (30%)	82 (45%)	–	31 (50%)	201 (37%)
Any	207 (70 %)	101 (55%)	–	31 (50%)	339 (63%)
Anthracycline	163	51	–	24	238
Non-anthracycline	41	50	–	7	98
Unknown type	3	0	–	0	3
Unknown	40	4	24	0	68
Radiation					
None	71 (26%)	86 (47%)	–	23 (37%)	180 (35%)
Any	204 (74%)	98 (53%)	–	39 (63%)	341 (65%)
Unknown	60	3	24	0	87
Endocrine therapy					
None	123 (44%)	94 (51%)	–	22 (36%)	239 (45%)
Any	159 (56%)	89 (49%)	–	39 (64%)	287 (55%)
Tamoxifen	35	74	–	21	130
Other type	12	14	–	18	44
Unknown type	112	1	–	0	113
Unknown	53	4	24	1	82
Anti-HER2 therapy					
None	322 (96%)	184 (99%)	–	60 (97%)	566 (97%)
Any	13 (4%)	1 (1%)	–	2 (3%)	16 (3%)
Unknown	0	2	24	0	26

Overall, 77% of the tumours were ER-positive. The proportion of ER-positive tumours declined with age; it was 83%, 79% and 72% for patients diagnosed ≤ 39 years, 40–50 years and >50 years, respectively (*P* for trend = 0.01). Overall, 52% of the tumours were lymph node positive; 59% of ER-positive patients were node-positive vs 34% of ER-negative patients (*P* < 0.001).

**Fig. 1:**
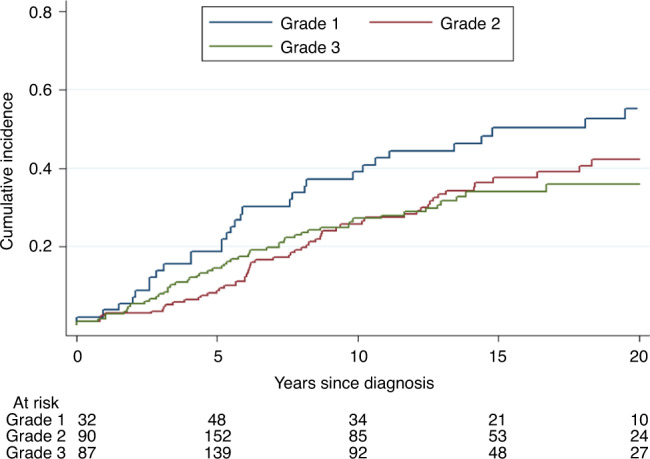
Cumulative incidence of breast cancer death according to grade (deaths due to other causes treated as competing events).

In the survival analyses, the total number of patients (excluding Norwegian cases) was 584 (Table 2). As expected, mortality increased with increasing tumour size and lymph node involvement. The crude survival for Grade 1 cancers was poorer than that for Grade 2 and 3 cancers, as shown in Fig. 1. In the adjusted analysis, this association was not statistically significant (Table 2). However, there were many fewer patients with Grade 1 tumours (*n* = 61) compared with Grade 2 and 3 tumours (*n* = 472).

**Table 2:** Risk of breast cancer-specific death in 584 Nordic carriers of *BRCA2* mutations according to key tumour characteristics, oophorectomy and treatment.

		Univariate disease-specific survival		Multivariate^a^ disease-specific survival	
		HR (95% CI)	*P* value	HR (95% CI)	*P* value
Year of diagnosis	Continuous	0.96 (0.94–0.98)	<0.001	0.98 (0.95–1.01)	0.16
Size >20–≤50 vs ≤20 mm	244 vs 311	1.73 (1.23–2.45)	<0.01	1.57 (1.07–2.32)	0.02
>50 vs ≤20 mm	29 vs 311	2.78 (1.49–5.19)	<0.01	2.65 (1.32–5.34)	0.01
Lymph node status (pos/neg)	314 vs 270	1.49 (1.06–2.09)	0.02	1.65 (1.04–2.62)	0.03
Grade 2 + 3 vs 1	472 vs 61	0.65 (0.40–1.05)	0.08	0.71 (0.43–1.16)	0.17
ER+ 0–5 y vs ER− ^b^	354 vs 126	0.42 (0.23–0.77)	<0.01	0.49 (0.26–0.93)	0.03
ER + 5+ y vs ER− ^c^	377 vs 126	1.60 (0.94–2.74)	0.09	1.91 (1.07–3.39)	0.03
Oophorectomy^d^	176 vs 408	0.39 (0.24–0.64)	<0.001	0.67 (0.38–1.20)	0.18
Mastectomy vs lumpectomy	369 vs 215	1.34 (0.92–1.93)	0.12	0.94 (0.59–1.51)	0.81
Adjuvant chemotherapy, any	383 vs 201	0.75 (0.54–1.05)	0.01	0.65 (0.43–1.00)	0.05
Radiation	404 vs 180	1.02 (0.72–1.45)	0.90	1.08 (0.70–1.65)	0.74
Endocrine therapy, any	345 vs 239	0.74 (0.53–1.04)	0.08	0.84 (0.53–1.32)	0.44
Mutation location, OCCRs + BCCRs vs “Other”^e^	391 vs 193	1.58 (1.04–2.41)	0.03	1.26 (0.76–2.09)	0.38

^a^Adjusted also for country, prophylactic mastectomy and unknown grade.

^b^Oestrogen-receptor positive up to 5 years after diagnosis vs oestrogen-receptor negative.

^c^Oestrogen-receptor positive 5 years or more after diagnosis vs oestrogen-receptor negative.

^d^Before or within 2 years from diagnosis.

^e^Other locations than ovarian cancer cluster regions (OCCRs) or breast cancer cluster regions (BCCRs).

**Fig. 2:**
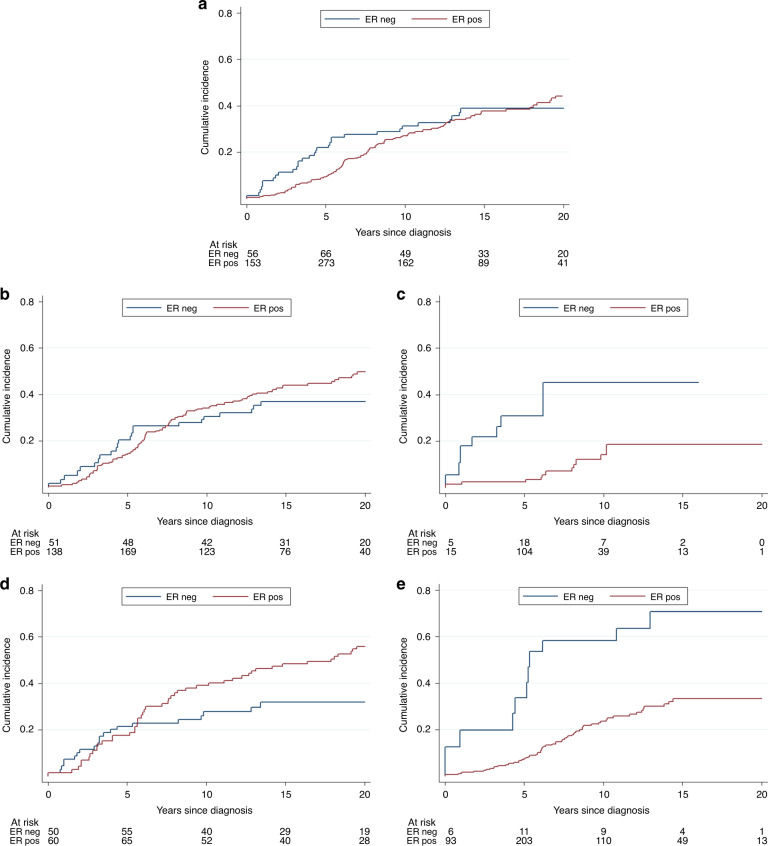
Cumulative incidence of breast cancer death according to ER status, stratified on oophorectomy and endocrine therapy (deaths due to other causes treated as competing events). **a** Without stratification. **b** Patients who did not undergo early oophorectomy. **c** Patients who underwent early oophorectomy. **d** Patients who did not receive endocrine therapy**. e** Patients who received endocrine therapy.

The 15-year survival was 60% for both ER-positive and ER-negative cancer patients (Fig. 2a). The multivariate hazard ratio for positive ER status and death was 1.15 (95% CI 0.74–1.79, *P* = 0.52); however, the hazard ratio was not constant over the follow-up period. During years 0–5, the HR for ER-positive status was 0.49 (95% CI 0.26–0.93, *P* = 0.03) and from year 5 onwards, it was 1.91 (95% CI 1.07–3.39, *P* = 0.03). Age at diagnosis was not associated with survival (data not shown), and menopausal status did not have a significant interaction with ER status (Table 3).

**Table 3:** Risk of breast cancer-specific death according to ER status (ER-positive vs ER-negative) for short (0–5 years) and long (5+ years) periods from diagnosis.

	0–5 years from diagnosis		5+ years from diagnosis	
	HR^a^ (95% CI; *P* value)	*P*-value interaction	HR^a^ (95% CI; *P* value)	*P*-value interaction
Age				
>50 y	0.46 (0.21–1.01; 0.05)		1.48 (0.75–2.91; 0.25)	
≤50 y	0.45 (0.22–0.91; 0.03)	0.98	1.91 (1.06–3.43; 0.03)	0.32
Endocrine treatment				
Yes	0.18 (0.08–0.43; <0.001)	0.01	0.72 (0.32–1.61; 0.43)	0.01
No	0.73 (0.33–1.63; 0.45)		2.36 (1.26–4.44; 0.01)	
Bilateral oophorectomy				
Yes	0.03 (0.00–0.29; <0.01)	0.01	0.61 (0.20–1.81; 0.37)	0.03
No	0.65 (0.33–1.27; 0.20)		1.99 (1.11–3.59; 0.02)	
Chemotherapy				
Yes	0.31 (0.14–0.68; <0.01)	0.10	1.41 (0.73–2.72; 0.37)	0.23
No	0.75 (0.31–1.81; 0.20)		2.40 (1.10–5.28; 0.02)	
*BRCA2* mutation location				
O + B^b^	0.63 (0.32–1.25; 0.19)	0.01	2.23 (1.21–4.10; 0.01)	0.01
Other	0.07 (0.02–0.36; <0.01)		0.56 (0.22–1.44; 0.24)	

Stratification by age, endocrine treatment, bilateral oophorectomy, chemotherapy and mutation location. Also shown are *P* values for interaction between ER status and the respective variable.

^a^Multivariate HR comparing ER+ with ER−, adjusting for other stratification variables in the table and also for year of diagnosis, size, lymph node status and prophylactic mastectomy.

^b^Ovarian cancer cluster regions (OCCRs) and breast cancer cluster regions (BCCRs).

Endocrine treatment was given to 55% of the patients (the majority took tamoxifen). The hazard ratio for endocrine therapy was 0.84 (95% CI 0.53–1.32, *P* = 0.44). After 5 years of follow-up, a positive ER status was adverse in women who did not have endocrine therapy (HR = 2.36; 95% CI 1.26–4.44, *P* = 0.01), but beneficial in women who received endocrine therapy (HR = 0.72; 95% CI 0.32–1.61, *P* = 0.43) (Table 3 and Fig. 2b, c). *P* = 0.01 for interaction with ER status.

Of the total group, 358 (59%) underwent bilateral oophorectomy, 11% before breast cancer diagnosis, 44% within 2 years from diagnosis and 45% 2 or more years after diagnosis. The proportion of women with oophorectomy ranged from 26% in Iceland (none of whom knew their mutation status) to 83% in Norway (all of whom knew their mutation status). In total, 32% of the subjects had an early oophorectomy (within the first 2 years of follow-up, including before diagnosis), and they were similar to women who did not undergo oophorectomy with respect to grade, tumour size, ER status and nodal status (data not shown), but they were diagnosed in more recent years. Early oophorectomy was associated with a modest non-significant reduction in the risk of death from breast cancer in the multivariate model (HR = 0.67; 95% CI 0.38–1.20, *P* = 0.18).

During the first 5 years, a positive ER status was beneficial in oophorectomised women (HR = 0.03; 95% CI 0.00–0.29, *P* < 0.01) (Table 3). An adverse association with a positive ER status 5 years from diagnosis was limited to women who did not have early oophorectomy (HR = 1.99; 95% CI 1.11–3.59, *P* = 0.02). This effect is shown in Fig. 2d, e. The association between ER status and survival differs greatly according to oophorectomy, in a similar way as for endocrine treatment (Fig. 2b, c). Of cases included in the survival analysis, 64% had either early oophorectomy or endocrine treatment. *P* for interaction between ER status and oophorectomy was 0.03 (Table 3).

About 63% of the patients received adjuvant chemotherapy (the majority received an anthracycline). After adjustment for other variables, women who received adjuvant chemotherapy had a marginally significantly lower risk of death than women who did not (HR = 0.65; 95% CI 0.43–1.00, *P* = 0.05) (Table 2). The effect of chemotherapy was not contingent on ER status, as *P* values for interaction with ER status were not statistically significant. Neither mastectomy nor radiation were associated with survival in the multivariate analysis.

The 608 Nordic patients carried 118 different pathogenic *BRCA2* variants (Supplementary Table 1). All Icelandic cases carried the same variant (995del5), which is located in a breast cancer cluster region (BCCR). Overall, 48% of all mutations were located in BCCRs, 18% were located in ovarian cancer cluster regions (OCCRs) and 34% were located outside those clusters (“Other”) (Supplementary Table 2). In the multivariate regression, location was not associated with survival, and inclusion of mutation location did not impact on estimates for other parameters. Interaction between mutation location and ER status was significant (Table 3).

## Discussion

In general, breast cancer patients with ER-positive breast tumours have better prognosis than patients with ER-negative cancers, although the benefit is mainly limited to the first 5 years after diagnosis.^13^ Low grade is also a well-established positive prognostic marker. In the current group of Nordic *BRCA2* carriers, these associations were not seen. An adverse effect of a positive ER status in *BRCA2* carriers was manifest 5 years from diagnosis. A favourable effect of positive ER status was present in *BRCA2* carriers shortly after diagnosis, but it was limited to 64% of cases who were not exposed to ovarian hormones, either because they had oophorectomy or received endocrine therapy that blocked oestrogen signalling.

A positive ER status was associated with higher prevalence of lymph node metastases at diagnosis (59%) than was a negative ER status (34%). This was reported earlier for Icelandic *BRCA2* carriers,^1^ in contrast with Icelandic non-carriers, where the percentage of lymph node-positive patients was lower for ER-positive than for ER-negative cancers.^1^ This implies that ER-positive breast cancers in *BRCA2* carriers are more prone to regional metastasis than are ER-positive tumours in the general population (we did not have data on Stage IV cancers to see if this was also true for distant metastases). Further, the proportion of ER-positive tumours was higher in carrier cases younger than 40 years at diagnosis (83%), than in those aged 51 or more (72%). This is in contrast to breast cancer patients in the general population, for whom the proportion of ER-positive tumours is the lowest at young ages (in a group of 27,000 Danish patients, it was 57% under the age of 40) and increases with advancing age at diagnosis.^13, 14^ We have shown previously that *BRCA2* patients have this paradoxical inverse relationship between ER-positive status and age, whereas *BRCA1* patients follow the conventional pattern.^14^

The impact of chemotherapy on death from breast cancer (HR = 0.65) was marginally significant after adjusting for stage and all other variables. We did not include a non-carrier control group, but in an earlier Icelandic study,^1^ the positive effect associated with adjuvant chemotherapy was considerably stronger for *BRCA2* carriers than for non-carriers. In another study, neoadjuvant chemotherapy was associated with a higher rate of pathological complete response in *BRCA1* and *BRCA2* carriers than in other patients with triple-negative breast cancer.^15^

The interaction between ER status and mutation location was significant. This suggests that the location of the mutation in the *BRCA2* gene might relate to how the tumour responds to ovarian hormones. However, this is the only study to date of *BRCA2* mutation location and survival, and the biological basis of the potential association is unknown. This finding needs confirmation in other studies.

The strengths of our study include a large group of *BRCA2* carriers for whom comprehensive clinical information was available. The current study is the largest to date. All Nordic countries have population-based health registries and national cancer registries. The use of unique personal identification numbers allows accurate record linkage to assess vital status, and loss to follow-up is negligible. For patients with blood samples drawn after diagnosis, we used left truncation in the survival analysis in order to avoid survivor bias.^16^ The patient populations and their treatment varied considerably between countries. The Norwegian group was limited to women diagnosed while under surveillance for breast cancer because of *BRCA2* mutations (prospective cases), which might explain why the Norwegian patients were, for the most part, diagnosed with early-stage cancers. On the other hand, only one of the 187 Icelandic patients had been mutation-tested before diagnosis, and for most of those patients, testing was based on stored paraffin samples or blood samples drawn at diagnosis. The year of diagnosis ranged from 1975 to 2018, so many patients were not given current treatment. The resulting variation in treatment confers a strength to our study, allowing contrasting effects of treatment vs no treatment to be evaluated. There was a large number of *BRCA2* variants in this Nordic study, but adjusting for variant location did not impact on the multivariate results. Currently, patients may be diagnosed with less advanced cancers due to more frequent screening and the introduction of MRI screening.

The definition of an ER-positive status varied with time and between countries. However, random misclassification would bias the HRs towards unity, and this variation is not likely to explain the associations observed in this study. We did not have information on the use of menopausal hormone treatment (MHT) after oophorectomy, but this is discouraged for breast cancer patients. We did not have information on menopausal status at diagnosis and used age 51 years as a surrogate for menopausal status. We could neither check any potential effects of the duration of endocrine therapy nor of ovarian suppression, as this information was not available.

In 2016, we reported that among carriers of an Icelandic *BRCA2* founder mutation, patients with ER-positive breast tumours had worse prognosis than patients with ER-negative cancers.^1^ Few of those carriers had undergone bilateral oophorectomies, as none knew their *BRCA2* mutation status. Those results were later confirmed in other studies.^17–20^ A similar inverse association between a positive ER status and poor prognosis has been reported in young women with breast cancer.^21–23^

The lack of observed association between low tumour grade and favourable prognosis was also atypical. In 2013, we reported for carriers of an Icelandic *BRCA2* founder mutation a worse prognosis associated with a low proliferation rate than with a high proliferation rate.^24^ We did not have information on S-phase fraction or Ki-67 in the present study, but evaluation criteria for histological grade include proliferation.^25^ Sotiriou C et al.^26^ identified 97 genes that were associated with histologic grade, and most of them were involved in cell cycle regulation and proliferation.

Our findings suggest that the BRCA2 protein could have a role in protecting the epithelial tissue of the breast against cancer-promoting effects of ovarian hormones. Cancers in hormonally responsive tissue with low amounts of the BRCA2 protein, would be abnormally stimulated by ovarian hormones. The current observation of a survival disadvantage 5 years from diagnosis in *BRCA2* carriers exposed to ovarian hormones supports such a possibility. Also, in support of this is the high proportion of ER-positive tumours among young *BRCA2* mutation carriers. Finally, the association between low grade and poorer prognosis in *BRCA2* carriers also points in this direction, as low grade is associated with more intact oestrogen signalling than is high grade.

These results suggest that the normal BRCA2 protein may have some function beyond that of preventing the initiation of cancer, and may hinder cancer progression mediated by sex hormones. In *BRCA2*-associated breast cancers, only half the amount of normal BRCA2 protein is expressed because only one functional copy of the gene is present, or there is loss of expression because of frequent loss of heterozygosity (around 50%)^27^ or gene silencing. The observed associations between ER status, survival and exposure to ovarian hormones may relate to the tissue specificity of cancers in *BRCA2* mutation carriers; mutation carriers have dramatic increases in susceptibility to both female breast and ovarian cancer, and breast cancer in males. All of the patients in the current study had germline *BRCA2* mutations, and it will be important to see if these relationships exist in breast cancer patients with somatic but no germline mutations.

The patients in the present study with ER-positive tumours benefitted from oophorectomies and endocrine therapy. However, half of the cases were diagnosed in 1975 through 2000 and 37% of patients did not receive chemotherapy. Therefore, we cannot conclude whether oophorectomies confer an additional advantage for *BRCA2* carriers who are treated according to current recommendations.

In summary, we find that ER-positive tumours have a poor long-term prognosis in *BRCA2* carriers, but blocking exposure to female hormones in the form of bilateral oophorectomy or endocrine therapy appears to mitigate this effect. *BRCA2* mutation carriers may be more sensitive to ovarian hormones than other breast cancer patients. Chemotherapy had a marginally significant effect, retained after adjusting for prognostic factors and other treatment. This study highlights the value of a woman knowing her *BRCA2* carrier status at the time her treatment is planned.

## Supplementary information

Supplementary Tables

## Data Availability

The datasets generated and/or analysed during the current study are not publicly available, due to protection of the privacy of *BRCA2* mutation carrier patients, but are available from the corresponding author on reasonable request.

## References

[1] Jonasson JG, Stefansson OA, Johannsson OT, Sigurdsson H, Agnarsson BA, Olafsdottir GH (2016). Oestrogen receptor status, treatment and breast cancer prognosis in Icelandic BRCA2 mutation carriers. Br. J. Cancer.

[2] IMH Soenderstrup, Laenkholm AV, Jensen MB, Eriksen JO, Gerdes AM, TVO Hansen (2018). Clinical and molecular characterization of BRCA-associated breast cancer: results from the DBCG. Acta Oncol..

[3] Evans DG, Harkness EF, Howell A, Wilson M, Hurley E, Holmen MM (2016). Intensive breast screening in BRCA2 mutation carriers is associated with reduced breast cancer specific and all cause mortality. Hered. Cancer Clin. Pract..

[4] Nilsson MP, Hartman L, Idvall I, Kristoffersson U, Johannsson OT, Loman N (2014). Long-term prognosis of early-onset breast cancer in a population-based cohort with a known BRCA1/2 mutation status. Breast Cancer Res. Treat..

[5] Nilsson MP, Hartman L, Kristoffersson U, Johannsson OT, Borg A, Henriksson K (2014). High risk of in-breast tumor recurrence after BRCA1/2-associated breast cancer. Breast Cancer Res. Treat..

[6] Winter C, Nilsson MP, Olsson E, George AM, Chen Y, Kvist A (2016). Targeted sequencing of BRCA1 and BRCA2 across a large unselected breast cancer cohort suggests that one-third of mutations are somatic. Ann. Oncol..

[7] Nilsson MP, Törngren T, Henriksson K, Kristoffersson U, Kvist A, Silfverberg B (2018). BRCAsearch: written pre-test information and BRCA1/2 germline mutation testing in unselected patients with newly diagnosed breast cancer. Breast Cancer Res. Treat..

[8] Rebbeck TR, Mitra N, Wan F, Sinilnikova OM, Healey S, McGuffog L (2015). Association of type and location of BRCA1 and BRCA2 mutations with risk of breast and ovarian cancer. JAMA.

[9] Royston P. “PTREND: Stata module for trend analysis for proportions”, Statistical Software Components S426101, (Boston College Department of Economics, 2014).

[10] Coviello V, Boggess M (2004). Cumulative incidence estimation in the presence of competing risks. Stata J..

[11] Lambert PC, Royston P (2009). Further development of flexible parametric models for survival analysis. Stata J..

[12] BAM Heemskerk-Gerritsen, Seynaeve C, van Asperen CJ, MGEM Ausems, Collée JM, van Doorn HC (2015). Breast cancer risk after salpingo-oophorectomy in healthy BRCA1/2 mutation carriers: revisiting the evidence for risk reduction. J. Natl Cancer Inst..

[13] Bentzon N, Düring M, Rasmussen BB, Mouridsen H, Kroman N (2008). Prognostic effect of estrogen receptor status across age in primary breast cancer. Int. J. Cancer.

[14] Foulkes WD, Metcalfe K, Sun P, Hanna WM, Lynch HT, Ghadirian P (2004). Estrogen receptor status in BRCA1- and BRCA2-related breast cancer: the influence of age, grade, and histological type. Clin. Cancer Res..

[15] Hahnen E, Lederer B, Hauke J, Loibl S, Kröber S, Schneeweiss A (2017). Germline mutation status, pathological complete response, and disease-free survival in triple-negative breast cancer: secondary analysis of the GeparSixto randomized clinical trial. JAMA Oncol..

[16] Azzato EM, Greenberg D, Shah M, Blows F, Driver KE, Caporaso NE (2009). Prevalent cases in observational studies of cancer survival: do they bias hazard ratio estimates?. BJC.

[17] Schmidt M. K., van den Broek A. J., Tollenaar R. A., Smit VTH, Westenend P. J., Brinkhuis M., et al. Breast cancer survival of BRCA1/BRCA2 mutation carriers in a hospital-based cohort of young women. *J. Natl Cancer Inst.* **109**, 1–10 (2017). 10.1093/jnci/djw329 28376189

[18] Copson ER, Maishman TC, Tapper WJ, Cutress RI, Greville-Heygate S, Altman DG (2018). Germline BRCA mutation and outcome in young-onset breast cancer (POSH): a prospective cohort study. Lancet Oncol..

[19] Metcalfe K, Lynch HT, Foulkes WD, Tung N, Olopade OI, Eisen A (2019). Oestrogen receptor status and survival in women with BRCA2-associated breast cancer. BJC.

[20] Vocka M, Zimovjanova M, Bielcikova Z, Tesarova P, Petruzelka L, Mateju M (2019). Estrogen receptor status oppositely modifies breast cancer prognosis in BRCA1/BRCA2 mutation carriers versus non-carriers. Cancers (Basel)..

[21] Liu Y-R, Jiang Y-Z, Yu K-D, Shao Z-M (2015). Different patterns in the prognostic value of age for breast cancer-specific mortality depending on hormone receptor status: a SEER population-based analysis. Ann. Surg. Oncol..

[22] Sopik V, Sun P, Narod SA (2017). The prognostic effect of estrogen receptor status differs for younger versus older breast cancer patients. Breast Cancer Res. Treat..

[23] Johansson ALV, Trewin CB, Hjerkind KV, Ellingjord-Dale M, Johannesen TB, Ursin G (2019). Breast cancer-specific survival by clinical subtype after 7 years follow-up of young and elderly women in a nationwide cohort. Int. J. Cancer.

[24] Tryggvadottir L, Olafsdottir EJ, Olafsdottir GH, Sigurdsson H, Johannsson OT, Bjorgvinsson E (2013). Tumour diploidy and survival in breast cancer patients with BRCA2 mutations. Breast Cancer Res. Treat..

[25] Rakha EA, Reis-Filho JS, Baehner F, Dabbs DJ, Decker T, Eusebi V (2010). Breast cancer prognostic classification in the molecular era: the role of histological grade. Breast Cancer Res..

[26] Sotiriou C, Wirapati P, Loi S, Harris A, Fox S, Smeds J (2006). Gene expression profiling in breast cancer: understanding the molecular basis of histologic grade to improve prognosis. J. Natl Cancer Inst..

[27] Maxwell KN, Wubbenhorst B, Wenz BM, De Sloover D, Pluta J, Emery L (2017). BRCA locus-specific loss of heterozygosity in germline BRCA1 and BRCA2 carriers. Nat. Commun..

