# Supplementary material for: Breast cancer survival in Nordic BRCA2 mutation carriers—unconventional association with oestrogen receptor status
Source: Br J Cancer. 2020 Sep 17;123(11):1608–15. doi: 10.1038/s41416-020-01056-4 (PMC7686356; doi:10.1038/s41416-020-01056-4)
Supplement: Supplementary file 1 — Supplementary Tables [file 41416_2020_1056_MOESM1_ESM.docx]

**Supplementary Table 1**

List of pathogenic mutations in 608 Nordic *BRCA2* mutation carriers, according to country

| **HGVS cDNA (NM_000059.3)** | **BIC cDNA (U43746)** | **DK** | **Ic** | **NO** | **SW** | **Total** |
| --- | --- | --- | --- | --- | --- | --- |
| c.1-?_10257+?del | del exon 2-27 | 1 |  |  |  | 1 |
| c.37G>T | 265G>T | 5 |  |  |  | 5 |
| c.68-?_316+?del | del exon 3 |  |  | 1 |  | 1 |
| c.145G>T | 373G>T | 8 |  |  |  | 8 |
| c.212dup | 440insA | 1 |  |  |  | 1 |
| c.289G>T | 517G>T | 1 |  |  |  | 1 |
| c.316+1G>T | IVS3+1G>T | 1 |  | 1 |  | 2 |
| c.316+5G>A | IVS3+5G>A | 1 |  |  |  | 1 |
| c.469_470del | 697delAA | 7 |  |  |  | 7 |
| c.516G>A | 744G>A | 7 |  |  |  | 7 |
| c.583del | 811delT | 1 |  |  |  | 1 |
| c.606dup | 824insC | 1 |  |  |  | 1 |
| c.631+4A>G | IVS7+4A>G | 2 |  |  |  | 2 |
| c.771_775del | 999delTCAAA | 2 | 187 |  | 1 | 190 |
| c.880G>T | 1108G>T | 1 |  |  |  | 1 |
| c.1233dup | 1461insA | 2 |  |  |  | 2 |
| c.1296_1297del | 1524delGA | 1 |  |  |  | 1 |
| c.1310_1313del | 1538delAAGA | 23 |  |  |  | 23 |
| c.1572del | 1800delG | 1 |  |  |  | 1 |
| c.1670_1683del | 1898del14 | 1 |  |  |  | 1 |
| c.1776_1776del | 2001delTTAT | 1 |  |  |  | 1 |
| c.1796_1800del | 2024delCTTAT |  |  | 3 | 1 | 4 |
| c.1813del | 2041delA | 10 |  |  |  | 10 |
| c.1813dup | 2041insA | 1 |  |  |  | 1 |
| c.2047_2050del | 2275delTCTC |  |  | 1 |  | 1 |
| c.2099T>A | 2327T>A | 1 |  |  |  | 1 |
| c.2245_2246ins20 | 2473ins20 | 1 |  |  |  | 1 |
| c.2376C>G | 2604C>G | 1 |  |  | 1 | 2 |
| c.2450del | 2678delA | 1 |  |  |  | 1 |
| c.2748T>A | 2976T>A | 2 |  |  |  | 2 |
| c.2808_2811del | 3036delACAA | 7 |  | 5 |  | 12 |
| c.2830A>T | 3058A>T | 8 |  | 1 | 4 | 13 |
| c.2899_2900del | 3127delCT | 1 |  |  |  | 1 |
| c.2979G>A | 3207G>A | 1 |  |  |  | 1 |
| c.3530_3533del | 3758delACAG | 3 |  |  |  | 3 |
| c.3545_3546del | 3773delTT | 1 |  |  |  | 1 |
| c.3599_3600del | 3827delGT | 1 |  |  |  | 1 |
| c.3847_3848del | 4075delGT | 16 |  | 4 | 2 | 22 |
| c.3860del | 4088delA | 3 |  |  |  | 3 |
| c.4037_4038del | 4265delCT |  |  |  | 2 | 2 |
| c.4258del | 4486delG | 3 |  |  | 15 | 18 |
| c.4284dup | 4512insT | 1 |  |  |  | 1 |
| c.4633del | 4861delC | 1 |  |  |  | 1 |
| c.4780del | 5008del | 1 |  |  |  | 1 |
| c.5130_5133del | 5358delTGTA | 4 |  |  |  | 4 |
| c.5164_5165del | 5392delAG |  |  |  | 1 | 1 |
| c.5213_5216del | 5441delCTTA | 1 |  |  | 2 | 3 |
| c.5217_5221del | 5445delTTTAA |  |  |  | 1 | 1 |
| c.5217_5223del | 5445delTTTAAGT |  |  | 1 | 1 | 2 |
| c.5219del | 5447delT | 1 |  |  | 1 | 2 |
| c.5238dup | 5466insT | 1 |  |  |  | 1 |
| c.5351del | 5579delA | 3 |  |  |  | 3 |
| c.5352del | 5580delC | 1 |  |  |  | 1 |
| c.5576_5579del | 5804delTTAA | 2 |  |  |  | 2 |
| c.5635G>T | 5863G>T |  |  |  | 1 | 1 |
| c.5645C>A | 5873C>A | 1 |  |  |  | 1 |
| c.5682C>G | 5910C>G | 1 |  |  |  | 1 |
| c.5754_5755del | 5982delTA | 5 |  |  |  | 5 |
| c.5857G>T | 6085G>T | 3 |  |  |  | 3 |
| c.5946del | 6174delT | 4 |  |  | 1 | 5 |
| c.6058G>T | 6286G>T | 1 |  |  |  | 1 |
| c.6059_6062del | 6287delAACA |  |  | 2 |  | 2 |
| c.6065C>G | 6293C>G |  |  |  | 6 | 6 |
| c.6082_6086del | 6310delGAAGA | 2 |  |  |  | 2 |
| c.6267_6269delinsC | 6495delGCAinsC | 1 |  |  | 1 | 2 |
| c.6373del | 6601delA | 27 |  |  |  | 27 |
| c.6443_6444del | 6671delCT | 3 |  |  |  | 3 |
| c.6444del | 6672delT |  |  |  | 1 | 1 |
| c.6486_6489del | 6714delACAA | 16 |  | 1 | 1 | 18 |
| c.6490_6492delinsGACT | 6718delCAGinsGACT | 3 |  |  |  | 3 |
| c.6601del | 6829delT | 1 |  |  |  | 1 |
| c.6641dup | 6869insC |  |  |  | 1 | 1 |
| c.6842-?_8331+?dup | exon 12-18dup | 2 |  |  |  | 2 |
| c.6901G>T | 7129G>T |  |  |  | 1 | 1 |
| c.6998dup | 7226insT |  |  |  | 1 | 1 |
| c.7007G>A | 7235G>A | 2 |  |  | 1 | 3 |
| c.7008-1G>A | IVS13-1G>A | 8 |  |  |  | 8 |
| c.7025_7026del | 7253delAA | 1 |  |  |  | 1 |
| c.7069_7070del | 7297delCT | 9 |  | 1 |  | 10 |
| c.7124T>G | 7352T>G |  |  |  | 1 | 1 |
| c.7177dup | 7405insA | 1 |  |  |  | 1 |
| c.7558C>T | 7786C>T |  |  | 1 | 1 | 2 |
| c.7617+1G>A | IVS15+1G>A | 29 |  |  |  | 29 |
| c.7757G>A | 7985G>A | 1 |  |  |  | 1 |
| c.7856G>A | 8085G>A | 1 |  |  |  | 1 |
| c.7872_7873del | 8100delTA | 1 |  |  |  | 1 |
| c.7878G>C | 8106G>C | 6 |  |  | 1 | 7 |
| c.7879A>T | 8107A>T |  |  |  | 1 | 1 |
| c.7913_7917del | 8141delTTCCT | 6 |  |  |  | 6 |
| c.7976G>A | 8204G>A | 1 |  |  |  | 1 |
| c.7980T>G | 8208T>G | 1 |  |  |  | 1 |
| c.7987G>A | 8215G>A | 3 |  |  |  | 3 |
| c.7988A>T | 8216A>T | 8 |  |  |  | 8 |
| c.8023A>G | 8251A>G |  |  |  | 2 | 2 |
| c.8165C>G | 8393C>G | 1 |  |  |  | 1 |
| c.8364G>A | 8592G>A | 1 |  |  |  | 1 |
| c.8474del | 8702delC | 2 |  |  |  | 2 |
| c.8536G>T | 8764G>T | 2 |  |  |  | 2 |
| c.8575del | 8803delC | 6 |  |  | 2 | 8 |
| c.8632+1G>T | IVS20+1G>T | 1 |  |  |  | 1 |
| c.8639_8640del | 8867delCA | 1 |  |  |  | 1 |
| c.8730del | 8958delT | 1 |  |  |  | 1 |
| c.8754+1G>A | IVS21+1G>A | 2 |  |  |  | 2 |
| c.8821C>T | 9049C>T | 2 |  |  |  | 2 |
| c.8931T>A | 9159T>A |  |  |  | 2 | 2 |
| c.8953+1G>T | IVS22+1G>T | 6 |  |  | 1 | 7 |
| c.9016_9017del | 9244delTA | 2 |  |  |  | 2 |
| c.9097dup | 9326insA | 3 |  |  |  | 3 |
| c.9106C>T | 9334C>T | 3 |  |  |  | 3 |
| c.9118-2A>G | IVS23-2A>G |  |  | 1 | 1 | 2 |
| c.9253dup | 9481insA | 2 |  |  |  | 2 |
| c.9382C>T | 9610C>T | 4 |  |  |  | 4 |
| c.9403del | 9631delC |  |  |  | 1 | 1 |
| c.9408del | 9636delT |  |  |  | 1 | 1 |
| c.9418_9430del13 | 9646del13 | 1 |  |  |  | 1 |
| c.9523G>T | 9751G>T |  |  | 1 |  | 1 |
| c.9580_9581del | 9808delCC | 1 |  |  | 1 | 2 |
| c.9699_9702del | 9927delTATG | 1 |  |  |  | 1 |
|  |  | 335 | 187 | 24 | 62 | 608 |

**Supplementary Table 2**

Distribution of mutations in Nordic *BRCA2* carrier cases according to mutation location and country

|  | OCCRs | BCCRs | “Other”* | Total |
| --- | --- | --- | --- | --- |
| Denmark | 71 (21%) | 96 (29%) | 168 (50%) | 335 |
| Iceland | 0 (0%) | 187 (100%) | 0 (0%) | 187 |
| Norway | 6 (25%) | 5 (21%) | 13 (54%) | 24 |
| Sweden | 31 (50%) | 6 (10%) | 25 (40%) | 62 |
| Total | 108 (18%) | 294 (48%) | 206 (34%) | 608 |

*Other locations than OCCRs or BCCRs
